# Atherogenic index of plasma and risk of hypertensive disorders of pregnancy in women with gestational diabetes mellitus: a two-center cohort study

**DOI:** 10.1038/s41440-026-02684-8

**Published:** 2026-05-19

**Authors:** Xueqi Bai, Qingyi Zhu, Haidong Wang, Runrun Hao, Shanshan Wang, Sutong Kan, Lina Zhang, Chun Zhao, Zhonghua Shi

**Affiliations:** 1 Changzhou Maternal and Child Health Care Hospital, Changzhou Medical Center, Nanjing Medical University, Changzhou, Jiangsu China; 2 Changzhou Key Laboratory of Maternal and Child Health Medicine, Changzhou, Jiangsu China; 3 Women’s Hospital of Nanjing Medical University, Nanjing Women and Children’s Healthcare Hospital, Nanjing, Jiangsu China; 4 The Affiliated Huai’an No.1 People’s Hospital of Nanjing Medical University, Huai’an, Jiangsu China

**Keywords:** Atherogenic index of plasma, Dyslipidemia, Hypertensive disorders of pregnancy, Gestational diabetes mellitus, Preeclampsia

## Abstract

Hypertensive disorders of pregnancy (HDPs) are major contributors to maternal and neonatal morbidity and are closely linked to metabolic disturbances. Women with gestational diabetes mellitus (GDM) exhibit significant lipid abnormalities, yet whether atherogenic lipid indices predict hypertensive complications in this population remains unclear. This study evaluated the predictive value of the atherogenic index of plasma (AIP) and cumulative AIP for HDPs and neonatal outcomes in women with GDM. In this two-center retrospective cohort study, a total of 3967 women with GDM were included. AIP was calculated as log10 (TG/HDL-C). Cumulative AIP was estimated as the mean AIP values measured during the second and third trimesters multiplied by the corresponding exposure time. Adverse outcomes were identified using generalized linear models with *P* for trend <0.05. Associations between AIP indices and outcomes were evaluated using regression models, dose–response analyses, and subgroup analyses. Predictive performance was assessed using receiver operating characteristic analysis. Both AIP and cumulative AIP were significantly associated with HDPs, including preeclampsia and preeclampsia with severe features, as well as neonatal intensive care unit (NICU) admission (all *P* < 0.05). Incorporating AIP into clinical models improved discrimination for preeclampsia (AUC 0.699 vs. 0.780, *P* = 0.025), severe preeclampsia (AUC 0.729 vs. 0.874, *P* = 0.002), and NICU admission (AUC 0.588 vs. 0.643, *P* = 0.029). Cumulative AIP produced similar improvements. In conclusion, elevated AIP and cumulative AIP are independently associated with increased risks of HDPs and NICU admission in women with GDM and may serve as practical cardiometabolic biomarkers for risk stratification.

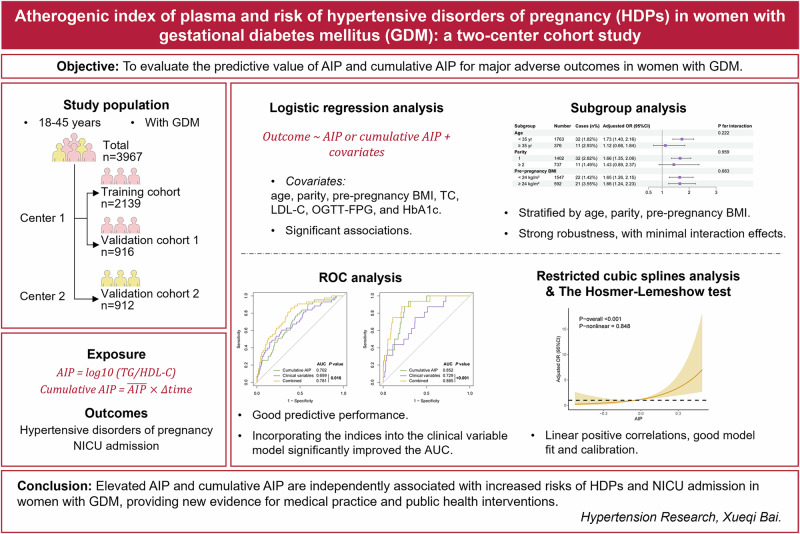

## Introduction

Gestational diabetes mellitus (GDM) is a common metabolic disorder during pregnancy and is increasingly recognized as an important contributor to adverse cardiovascular and obstetric outcomes. Women with GDM are at substantially higher risk of developing hypertensive disorders of pregnancy (HDPs), including preeclampsia (PE), which remains a leading cause of maternal and neonatal morbidity worldwide [1]. The global prevalence of GDM has risen to approximately 14% over the past decade, largely driven by increasing rates of obesity and metabolic dysfunction among women of reproductive age [2]. Emerging evidence indicates a close and bidirectional relationship between GDM and HDPs. Women with GDM have a 1.24–1.46-fold higher risk of PE, while the vascular and endothelial dysfunction associated with PE may further aggravate insulin resistance and metabolic dysregulation during pregnancy [3, 4]. These interactions highlight the importance of identifying early cardiometabolic markers that may help predict hypertensive complications in this high-risk population.

The atherogenic index of plasma (AIP), defined as the logarithmic transformation of the triglyceride-to-high-density lipoprotein cholesterol (TG/HDL-C) ratio, has emerged as a reliable indicator of atherogenic dyslipidemia and cardiovascular risk [5, 6]. AIP reflects the balance between pro-atherogenic TG-rich lipoproteins and protective HDL particles and is closely associated with insulin resistance, endothelial dysfunction, and metabolic syndrome [7]. Recent studies have shown that elevated AIP in early pregnancy is associated with an increased risk of developing GDM, suggesting that AIP may serve as a simple and cost-effective biomarker for metabolic risk stratification during pregnancy [8]. However, whether AIP can further predict HDPs and adverse neonatal outcomes among women already diagnosed with GDM remains unclear.

Most previous studies evaluating AIP in pregnancy have relied on single-time-point measurements. Pregnancy is characterized by profound physiological changes in lipid metabolism, with progressive increases in TGs and other lipoproteins across gestation [9]. Therefore, cumulative exposure to AIP may better reflect the overall metabolic burden affecting maternal vascular function during pregnancy. Cumulative AIP, which integrates repeated AIP measurements over time, has shown value in predicting metabolic diseases such as diabetes in the general population [10]. Nevertheless, its clinical relevance in pregnant populations, particularly for predicting HDPs and related neonatal outcomes, has not been investigated.

Therefore, using data from two maternal and child health centers, we evaluated the associations of both single-time-point AIP and cumulative AIP during pregnancy with HDPs and neonatal intensive care unit (NICU) admission among women with GDM. By examining longitudinal lipid exposure during pregnancy, this study aims to clarify the role of AIP in the development of HDPs in GDM and to assess whether dynamic lipid indices may improve risk stratification in this high-risk population.

Point of view

- **Clinical relevance**
  AIP and cumulative AIP may serve as simple lipid-related markers to identify women with GDM at higher risk of hypertensive complications and NICU admission.
- **Future direction**
  Prospective multicenter studies are needed to validate optimal AIP-based thresholds and to determine whether AIP-guided surveillance or intervention can improve maternal and neonatal outcomes.
- **Consideration for the Asian population**
  AIP may be particularly useful in Asian populations, where metabolic risk can be substantial despite relatively low BMI.

## Methods

### Study design and participants

**Fig. 1:**
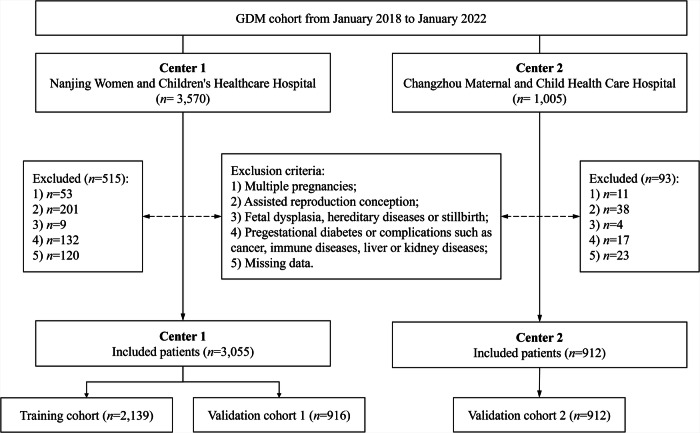
Flow chart of study participants

This was a large observational cohort study conducted at Women’s Hospital of Nanjing Medical University (Center 1) and Changzhou Maternal and Child Health Care Hospital (Center 2). Data from patients with GDM admitted from January 2018 to January 2022 were retrospectively analyzed. All eligible patients aged 18–45 years who met the diagnostic criteria for GDM were included. GDM was defined via a standardized 75-g oral glucose tolerance test (OGTT) at 24–28 weeks of gestation if any of the following thresholds were met: fasting plasma glucose measured at the 0-h time point (OGTT-FPG) ≥ 5.1 mmol/L, 1-h plasma glucose (OGTT-1 h) ≥10.0 mmol/L, or 2-h plasma glucose (OGTT-2 h) ≥8.5 mmol/L [11]. The exclusion criteria were as follows: (1) multiple pregnancies; (2) assisted reproduction conception; (3) fetal dysplasia, hereditary diseases or stillbirth; (4) pregestational diabetes mellitus or complications such as cancer, immune diseases, liver or kidney diseases; (5) missing baseline data. Finally, 3967 women were enrolled: 3055 from Center 1 and 912 from Center 2. The participants from Center 1 were randomly divided into a training cohort (70%, *n* = 2139) and a validation cohort (30%, *n* = 916) via computer-generated random numbers. An independent cohort from Center 2 (*n* = 912) served as the external validation set (Validation cohort 2) to evaluate model generalizability. The sample selection process is depicted in Fig. 1.

The study was implemented in accordance with the Declaration of Helsinki and was authorized by the Medical Ethics Committee of Nanjing Women and Children’s Healthcare Hospital (2022KY-165-01) and the Ethics Committee of Changzhou Maternal and Child Health Care Hospital (2022[106]). The need for informed consent was waived because of its retrospective nature, and information related to patient identity was anonymized.

### Data collection

Clinical data, including demographic and medical characteristics, laboratory values, maternal complications, and neonatal disease features, were collected from the Hospital Information System. The demographic characteristics included age, pre-pregnancy body mass index (BMI) and parity. Venous blood samples were collected after overnight fasting in the second trimester (20–24 weeks of gestation) and the third trimester (30–34 weeks of gestation). The laboratory test results included the levels of glycated hemoglobin A1c (HbA1c), total cholesterol (TC), TG, low-density lipoprotein cholesterol (LDL-C), and HDL-C.

Maternal diseases included HDPs (PE and PE with severe features), oligohydramnios, polyhydramnios, intrahepatic cholestasis of pregnancy (ICP), fetal distress, and postpartum hemorrhage. Neonatal outcomes included transient tachypnoea of the newborn (TTN), neonatal respiratory distress syndrome (RDS), asphyxia of the newborn, neonatal hypoglycemia, neonatal hyperbilirubinemia, large for gestational age (LGA), small for gestational age (SGA), and admission to NICU. The outcomes were defined using the corresponding ICD-10 codes in the medical reports.

### Calculation of AIP and cumulative AIP

The AIP index was calculated using the following formula: log10 [TG (mmol/L)/HDL-C (mmol/L)] [12]. Cumulative AIP was estimated as the mean AIP values measured during the second (20–24 weeks) and third (30–34 weeks) trimesters multiplied by the corresponding exposure time, specifically as follows: (AIP _the second trimester_ + AIP _the third trimester_)/2 * time _(the third trimester – the second trimester)_ [10].

### Statistical analysis

The baseline characteristics of the study population were stratified by the AIP and cumulative AIP quartiles (Q1–Q4). Continuous variables are presented as the means with standard deviations and were compared using one-way analysis of variance when normally distributed. Categorical variables are summarized as counts with proportions and were compared using the χ^2^ test or Fisher’s exact test.

Generalized linear models with a binomial distribution and logit link were used to calculate the *P* for trend across quartiles of AIP and cumulative AIP for the GDM-related adverse pregnancy outcomes. Outcomes with a *P* for trend <0.05 were selected for subsequent analyses. Univariate and multivariable-adjusted logistic regression analyses were used to estimate the odds ratios (ORs) and 95% confidence intervals (CIs) for pregnancy outcomes. Three models were developed: Model 1 used unadjusted data; Model 2 was adjusted for age, pre-pregnancy BMI, and parity; and Model 3 was further adjusted for TC, LDL-C, OGTT-FPG, and HbA1c. Both AIP and cumulative AIP were included as continuous or categorical variables to assess their independent roles as risk factors for pregnancy outcomes in each model. Firth’s bias-reduced logistic regression was applied to address complete separation issues in the logistic regression model, where outcomes in the first quartile group were absent [13]. To further assess the linearity of the relationship between AIP and adverse pregnancy outcomes, restricted cubic spline (RCS) analysis was conducted after adjusting for the variables in Model 3, with four distribution knots at the 25th, 50th, 75th and 95th percentiles. The Hosmer–Lemeshow goodness-of-fit test was used to assess whether the models were correctly specified.

Subgroup analysis was performed after the participants were stratified by age (<35 or ≥35 years), parity (1 or ≥2), and pre-pregnancy BMI (<24 or ≥24 kg/m^2^) to identify the impact of these factors. Interactions across subgroups were tested using the likelihood ratio test.

Receiver operating characteristic (ROC) curve analysis was employed to evaluate the discriminatory ability and performance of the AIP and cumulative AIP in predicting adverse maternal and neonatal outcomes. The area under the ROC curve (AUC) was calculated and compared via DeLong’s test to evaluate the predictive value after incorporating AIP or cumulative AIP index into the baseline clinical model. For combined models, predicted probabilities from multivariable logistic regression models were used to generate ROC curves. The baseline clinical factors included age, pre-pregnancy BMI, parity, TC, LDL-C, OGTT-FPG, and HbA1c.

Statistical analysis was conducted using R software (version 4.2.0). Two-sided statistical significance was set at *P* < 0.05.

## Results

### Baseline characteristics of the study population

The baseline characteristics of the training cohort stratified by quartiles of AIP and cumulative AIP are shown in Tables 1 and 2. Compared with women in the lowest AIP quartile, those in higher quartiles were more likely to be older, multiparous, and to have higher pre-pregnancy BMI, OGTT-FPG, HbA1c, TC, and LDL-C levels. Infants born to women with higher AIP levels had lower gestational age at delivery and higher birth weight. Similar patterns were observed when participants were categorized according to cumulative AIP (Table 2). Comparisons between the training and validation cohorts are presented in Supplementary Table 1.

**Table 1:** Characteristics of the study population according to AIP quartiles

Characteristics	AIP quartiles				*P* value
	Q1 (*n* = 535)	Q2 (*n* = 535)	Q3 (*n* = 535)	Q4 (*n* = 534)	
Maternal characteristics					
Age, years (SD)	29.89 (3.86)	30.56 (3.95)	30.61 (4.03)	31.24 (4.19)	**<0.001**
Pre-pregnancy BMI, kg/m^2^ (SD)	22.53 (2.04)	23.07 (2.23)	23.43 (2.20)	23.89 (2.21)	**<0.001**
Parity, *n* (%)					**<0.001**
1	389 (72.71%)	361 (67.48%)	342 (63.93%)	310 (58.05%)	
≥2	146 (27.29%)	174 (32.52%)	193 (36.07%)	224 (41.95%)	
OGTT-FPG, mmol/L (SD)	4.70 (0.41)	4.76 (0.38)	4.81 (0.40)	4.83 (0.39)	**<0.001**
OGTT-1 h, mmol/L (SD)	8.85 (1.67)	8.91 (1.74)	8.93 (1.58)	9.02 (1.62)	0.376
OGTT-2 h, mmol/L (SD)	7.74 (1.55)	7.79 (1.46)	7.81 (1.47)	7.76 (1.47)	0.870
HbA1c, % (SD)	4.96 (0.30)	5.04 (0.31)	5.09 (0.33)	5.11 (0.32)	**<0.001**
HbA1c, mmol/mol (SD)	30.74 (3.24)	31.62 (3.34)	32.13 (3.61)	32.39 (3.44)	**<0.001**
Second-trimester TC, mmol/L (SD)	6.00 (0.88)	5.86 (0.92)	5.88 (0.96)	5.60 (1.00)	**<0.001**
Second-trimester LDL-C, mmol/L (SD)	2.92 (0.67)	3.04 (0.71)	3.12 (0.74)	2.91 (0.78)	**<0.001**
Neonatal characteristics					
Gestational age, weeks (SD)	39.47 (1.11)	39.42 (1.20)	39.35 (1.22)	39.14 (1.34)	**<0.001**
Birth weight, g (SD)	3382.52 (391.47)	3389.38 (424.44)	3414.06 (433.86)	3445.41 (470.50)	0.072

The bold *P* value indicates statistical significance

Data are presented as mean (standard deviation) or *n* (%)

*AIP* atherogenic index of plasma, *BMI* body mass index, *FPG* fasting plasma glucose, *HbA1c* glycated hemoglobin, *LDL-C* low-density lipoprotein-cholesterol, *OGTT* oral glucose tolerance test, *Q* quartiles, *TC* total cholesterol

**Table 2:** Characteristics of the study population according to cumulative AIP quartiles

Characteristics	Cumulative AIP quartiles				*P* value
	Q1 (*n* = 535)	Q2 (*n* = 535)	Q3 (*n* = 535)	Q4 (*n* = 534)	
Maternal characteristics					
Age, years (SD)	29.96 (3.82)	30.44 (4.02)	30.83 (4.03)	31.07 (4.19)	**<0.001**
Pre-pregnancy BMI, kg/m^2^ (SD)	22.58 (2.13)	22.95 (2.15)	23.50 (2.26)	23.89 (2.13)	**<0.001**
Parity, *n* (%)					**<0.001**
1	383 (71.59%)	374 (69.91%)	334 (62.43%)	311 (58.24%)	
≥ 2	152 (28.41%)	161 (30.09%)	201 (37.57%)	223 (41.76%)	
OGTT-FPG, mmol/L (SD)	4.69 (0.41)	4.77 (0.38)	4.82 (0.40)	4.83 (0.39)	**<0.001**
OGTT-1 h, mmol/L (SD)	8.85 (1.70)	8.89 (1.71)	8.96 (1.58)	9.01 (1.61)	0.340
OGTT-2 h, mmol/L (SD)	7.71 (1.56)	7.87 (1.44)	7.78 (1.47)	7.75 (1.47)	0.369
HbA1c, % (SD)	4.96 (0.30)	5.02 (0.29)	5.10 (0.32)	5.12 (0.33)	**<0.001**
HbA1c, mmol/mol (SD)	30.76 (3.25)	31.40 (3.17)	32.21 (3.51)	32.51 (3.65)	**<0.001**
Second-trimester TC, mmol/L (SD)	5.92 (0.90)	5.94 (0.89)	5.86 (0.96)	5.61 (1.02)	**<0.001**
Second-trimester LDL-C, mmol/L (SD)	2.88 (0.67)	3.08 (0.67)	3.12 (0.74)	2.92 (0.80)	**<0.001**
Neonatal characteristics					
Gestational age, weeks (SD)	39.53 (1.04)	39.43 (1.24)	39.31 (1.17)	39.12 (1.39)	**<0.001**
Birth weight, g (SD)	3378.45 (375.87)	3389.42 (426.62)	3403.21 (433.87)	3460.32 (479.53)	**0.010**

The bold *P* value indicates statistical significance

Data are presented as mean (standard deviation) or *n* (%)

*AIP* atherogenic index of plasma, *BMI* body mass index, *FPG* fasting plasma glucose, *HbA1c* glycated hemoglobin, *LDL-C* low-density lipoprotein-cholesterol, *OGTT* oral glucose tolerance test, *Q* quartiles, *TC* total cholesterol

### Associations of AIP and cumulative AIP with HDPs and NICU admission

The prevalence of GDM-related adverse outcomes across AIP and cumulative AIP quartiles is presented in Supplementary Tables 2, 3. Notably, the rates of PE, PE with severe features, and NICU admission were significantly higher in the Q2, Q3, and Q4 groups than in the Q1 group (*P* for trend ≤0.05). No significant differences were observed for the other outcomes, prompting us to focus on these three adverse outcomes for further analysis.

#### Associations between AIP and study outcomes

The associations of AIP with HDPs and neonatal outcomes were assessed using logistic regression models (Table 3). Positive associations between AIP and the rates of PE, PE with severe features, and NICU admission were observed in Model 1 (crude), Model 2 (adjusted for demographic factors) and Model 3 (adjusted for additional covariates, including TC, LDL-C, OGTT-FPG, and HbA1c). When AIP was categorized into quartiles, women in the higher AIP quartiles (Q2–Q4) had significantly greater risks for these outcomes than those in Q1 (*P* for trend <0.05).

**Table 3:** Associations of AIP with HDPs and neonatal outcomes

	Continuous	AIP quartiles				*P* for trend
		Q1	Q2	Q3	Q4	
PE						
Model 1	2.17 (1.64–2.86)	Ref.	4.05 (1.01–26.89)	6.64 (1.82–42.58)	10.37 (3.01–65.17)	**<0.001**
Model 2	2.01 (1.51–2.69)	Ref.	3.58 (0.89–23.84)	5.63 (1.53–36.30)	8.11 (2.30–51.47)	**0.001**
Model 3	2.02 (1.50–2.73)	Ref.	3.50 (0.85–23.65)	5.73 (1.49–37.80)	7.98 (2.16–51.84)	**0.001**
PE with severe features						
Model 1	3.64 (2.40–5.74)	Ref.	1.00 (0.01–184.54)	11.10 (1.25–1458.98)	23.53 (3.06–3022.55)	**<0.001**
Model 2	3.49 (2.27–5.57)	Ref.	0.88 (0.01–162.36)	9.25 (1.04–1218.17)	17.67 (2.22–2285.02)	**<0.001**
Model 3	3.23 (2.05–5.40)	Ref.	1.09 (0.01–203.32)	12.88 (1.35–1722.49)	21.42 (2.51–2810.19)	**<0.001**
Admission to NICU						
Model 1	1.40 (1.12–1.74)	Ref.	1.26 (0.58–2.77)	1.69 (0.83–3.60)	2.41 (1.24–4.97)	**0.006**
Model 2	1.44 (1.14–1.80)	Ref.	1.31 (0.60–2.88)	1.79 (0.87–3.83)	2.64 (1.33–5.55)	**0.003**
Model 3	1.44 (1.14–1.81)	Ref.	1.39 (0.63–3.13)	2.01 (0.94–4.46)	2.81 (1.36–6.15)	**0.003**

The bold *P* value indicates statistical significance

Model 1 was unadjusted. Model 2 was Model 1 adjusted for age, pre-pregnancy BMI, and parity. Model 3 was Model 2 adjusted for TC, LDL-C, OGTT-FPG, and HbA1c. Firth’s bias-reduced logistic regression was applied due to complete separation in the first quartile group of PE with severe features

*AIP* atherogenic index of plasma, *HDPs* hypertensive disorders of pregnancy, *GDM* gestational diabetes mellitus, *NICU* neonatal intensive care unit, *PE* preeclampsia, *Q* quartiles

#### Associations between cumulative AIP and study outcomes

The continuous cumulative AIP was positively correlated with the three main adverse maternal and neonatal outcomes in all three models (*P* < 0.05, Table 4). When cumulative AIP was divided into quartiles, the risk of adverse outcomes in higher quartiles (Q2–Q4) remained significantly greater than that in Q1, with a clear trend toward increasing risk across quartiles (*P* for trend <0.05 in three models).

**Table 4:** Associations of cumulative AIP with HDPs and neonatal outcomes

	Continuous	Cumulative AIP quartiles				*P* for trend
		Q1	Q2	Q3	Q4	
PE						
Model 1	2.19 (1.66–2.88)	Ref.	3.53 (0.85–23.79)	6.64 (1.82–42.58)	10.91 (3.18–68.43)	**<0.001**
Model 2	2.06 (1.55–2.73)	Ref.	3.17 (0.76–21.42)	5.56 (1.51–35.87)	8.77 (2.51–55.43)	**<0.001**
Model 3	2.07 (1.54–2.78)	Ref.	3.35 (0.79–22.91)	5.88 (1.53–38.81)	8.82 (2.41–57.13)	**0.001**
PE with severe features						
Model 1	3.83 (2.52–6.06)	Ref.	3.01 (0.16–438.99)	5.02 (0.41–692.84)	27.72 (3.67–3550.76)	**<0.001**
Model 2	3.81 (2.46–6.18)	Ref.	2.74 (0.15–400.32)	4.16 (0.34–575.94)	21.38 (2.76–2750.99)	**<0.001**
Model 3	3.63 (2.26–6.18)	Ref.	3.74 (0.19–551.18)	5.97 (0.46–836.79)	26.73 (3.22–3485.64)	**<0.001**
Admission to NICU						
Model 1	1.32 (1.05–1.65)	Ref.	1.56 (0.73–3.47)	1.95 (0.95–4.22)	2.44 (1.22–5.19)	**0.011**
Model 2	1.35 (1.07–1.69)	Ref.	1.60 (0.75–3.56)	2.06 (0.99–4.51)	2.64 (1.30–5.71)	**0.007**
Model 3	1.36 (1.07–1.70)	Ref.	1.80 (0.83–4.08)	2.41 (1.12–5.45)	2.92 (1.38–6.54)	**0.005**

The bold *P* value indicates statistical significance

Model 1 was unadjusted. Model 2 was Model 1 adjusted for age, pre-pregnancy BMI, and parity. Model 3 was Model 2 adjusted for TC, LDL-C, OGTT-FPG, and HbA1c. Firth’s bias-reduced logistic regression was applied due to complete separation in the first quartile group of PE with severe features

*AIP* atherogenic index of plasma, *HDPs* hypertensive disorders of pregnancy, *GDM* gestational diabetes mellitus, *NICU* neonatal intensive care unit, *PE* preeclampsia, *Q* quartiles

The Hosmer–Lemeshow test was employed to calibrate the models for adverse pregnancy outcomes. The results indicated that all the models were well calibrated (all *P* > 0.05; Supplementary Tables 4, 5).

### Dose‒response relationships of AIP exposure with HDPs and NICU admission

As shown in Supplementary Fig. 1, we used RCS curves with 4 knots to flexibly model and visualize the relationships between AIP and maternal and neonatal outcomes. After adjusting for covariates, a linear positive correlation was observed for PE, PE with severe features, and NICU admission (all *P* values for non-linearity >0.05; *P* for overall <0.05). Similarly, a dose‒response relationship was evident between cumulative AIP and these adverse outcomes after adjusting for multiple covariates (all *P* values for non-linearity >0.05; *P* for overall <0.05; Supplementary Fig. 1).

### Subgroup analyses

Subgroup analyses were conducted to evaluate whether the associations between AIP (and cumulative AIP) and adverse outcomes differed across categories of maternal age, pre-pregnancy BMI, and parity (Supplementary Figs. 2, 3). No significant interactions were observed between AIP and these subgroup variables in relation to the risks of PE, PE with severe features, or NICU admission (all *P* for interaction >0.05). Similar results were observed for cumulative AIP, indicating that the associations between cumulative AIP and adverse outcomes were consistent across the examined subgroups.

### Predictive performance of AIP and cumulative AIP for HDPs and NICU admission

**Fig. 2:**
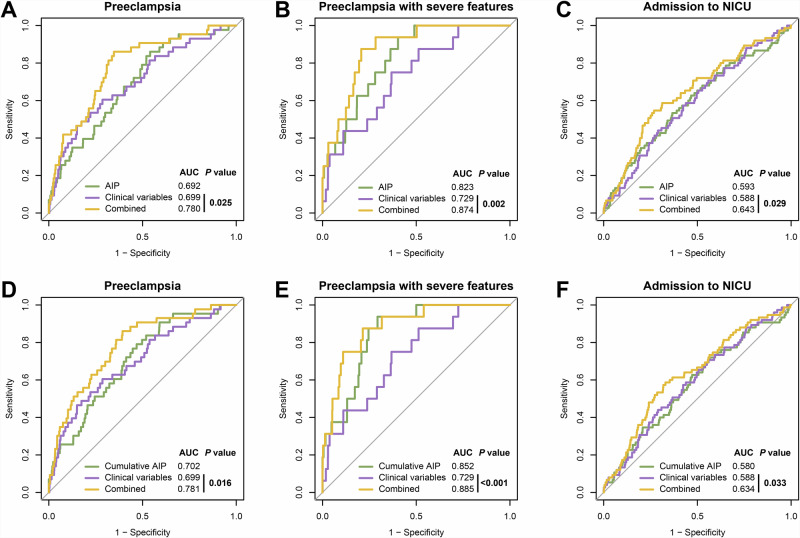
Predictive power of AIP and cumulative AIP for HDPs and neonatal outcomes. ROC curves of AIP for preeclampsia (**A**), preeclampsia with severe features (**B**), and admission to NICU (**C**). ROC curves of cumulative AIP for preeclampsia (**D**), preeclampsia with severe features (**E**), and admission to NICU (**F**). Clinical variables included age, pre-pregnancy BMI, parity, TC, LDL-C, OGTT-FPG, and HbA1c. AIP atherogenic index of plasma, AUC area under the curve, HDPs hypertensive disorders of pregnancy, GDM gestational diabetes mellitus, NICU neonatal intensive care unit

ROC curve analysis of the ability of the AIP to predict adverse outcomes is shown in Fig. 2 and Supplementary Table 6. AIP alone demonstrated moderate predictive ability for PE (AUC 0.692, 95% CI 0.617–0.767) and PE with severe features (AUC 0.823, 95% CI 0.774–0.902), but its utility for predicting NICU admission was limited (AUC 0.593, 95% CI 0.525–0.662). Importantly, incorporating AIP into the clinical model significantly improved the prediction of PE (from 0.699 [95% CI 0.616–0.783] to 0.780 [95% CI 0.714–0.847], *P* = 0.025), PE with severe features (from 0.729 [95% CI 0.611–0.848] to 0.874 [95% CI 0.808–0.940], *P* = 0.002) and NICU admission (from 0.588 [95% CI 0.523–0.653] to 0.643 [95% CI 0.578–0.709], *P* = 0.029), indicating that AIP provides additional predictive information beyond conventional clinical predictors. The Youden index was used to determine the optimal cutoff values of AIP for predicting PE, PE with severe features, and NICU admission. The corresponding thresholds and diagnostic performance are summarized in Supplementary Table 4.

The AUCs for cumulative AIP were 0.702 (95% CI 0.629–0.775) for PE, 0.852 (95% CI 0.783–0.920) for PE with severe features, and 0.580 (95% CI 0.513–0.647) for admission to NICU, respectively. Adding cumulative AIP to the clinical risk model significantly increased the model’s AUC for PE (from 0.699 [95% CI 0.616–0.783] to 0.781 [95% CI 0.714–0.849], *P* = 0.016), PE with severe features (from 0.729 [95% CI 0.611–0.848] to 0.855 [95% CI 0.814–0.957], *P* < 0.001), and admission to NICU (from 0.588 [95% CI 0.523–0.653] to 0.634 [95% CI 0.570–0.698], *P* = 0.033). A summary of the AUCs for each model is shown in Fig. 2 and Supplementary Table 7.

### Validation of predictive models

The baseline characteristics of participants in validation cohort 1 and validation cohort 2 stratified by quartiles of AIP and cumulative AIP are presented in Supplementary Tables 8, 9, 14, 15. In both validation cohorts, participants in higher AIP or cumulative AIP quartiles generally showed higher pre-pregnancy BMI, OGTT-FPG, HbA1c, TC, LDL-C levels, and neonatal birth weight, consistent with the trends observed in the training cohort.

In validation cohort 1, both AIP and cumulative AIP were significantly associated with adverse outcomes. Compared with those in the lowest quartile (Q1), women in the highest AIP quartile (Q4) had greater risks of PE (OR 6.87, 95% CI 1.88–44.25), PE with severe features (OR 7.35, 95% CI 1.31–138.73), and NICU admission (OR 4.38, 95% CI 1.50–16.09) after full adjustment (Supplementary Table 10). Similarly, cumulative AIP was associated with elevated risks for PE (OR 7.49, 95% CI 2.06–48.26), PE with severe features (OR 5.69, 95% CI 1.26–54.34), and NICU admission (OR 4.72, 95% CI 1.44–21.30) (Supplementary Table 11). The predictive model integrating the AIP score with clinical variables maintained improved performance over the clinical model alone. Models incorporating AIP with clinical variables improved the prediction of all three outcomes, as reflected by the increased AUC for PE (from 0.724 to 0.800, *P* = 0.029), PE with severe features (from 0.703 to 0.834, *P* = 0.023), and NICU admission (from 0.582 to 0.702, *P* = 0.040) (Supplementary Table 12). The models combining cumulative AIP with clinical variables retained predictive improvement over the baseline models (PE *P* = 0.016, PE with severe features *P* = 0.006, NICU admission *P* = 0.037) (Supplementary Table 13). All the models were well calibrated (Hosmer–Lemeshow test *P* > 0.05) (Supplementary Table 4 and Supplementary Table 5).

In external validation cohort 2, AIP and cumulative AIP remained significantly associated with PE, PE with severe features, and NICU admission (all *P* < 0.05) (Supplementary Tables 16, 17). Compared with baseline clinical models (Supplementary Table 18), models combining AIP with clinical risk factors showed improved predictive power for PE (AUC 0.762, *P* = 0.036), PE with severe features (AUC 0.785, *P* = 0.035) and NICU admission (AUC 0.695, *P* = 0.044). The addition of the cumulative AIP increased the predictive accuracy for all three outcomes (Supplementary Table 19). All the models showed adequate calibration (Hosmer–Lemeshow *P* > 0.05) (Supplementary Table 6 and Supplementary Table 7).

## Discussion

Our study demonstrated that both second-trimester AIP and cumulative AIP during pregnancy were independently associated with an increased risk of HDPs in women with GDM, particularly PE and PE with severe features, as well as NICU admission. Dose–response analyses revealed a progressive increase in risk with increasing AIP levels, supporting a potential role of sustained atherogenic lipid exposure in the development of vascular complications during pregnancy. When incorporated into clinical models, both AIP and cumulative AIP provided similar improvements in risk prediction. These findings highlight the potential role of AIP as an accessible cardiometabolic marker for identifying women with GDM who are at increased risk of hypertensive complications.

AIP reflects an atherogenic lipid profile characterized by elevated TG and reduced HDL-C and has been widely used as a marker of cardiometabolic risk in nonpregnant populations [14–16]. Previous studies have suggested that AIP correlates with insulin resistance and type 2 diabetes in nonpregnant populations, and elevated AIP has also been associated with the development of GDM [5, 8]. Our findings extend this concept by demonstrating that atherogenic lipid profiles are also associated with hypertensive complications in women with GDM. Epidemiological evidence indicates that most cases of PE are late-onset, accounting for ~87% of cases and typically occurring after 34 weeks of gestation [17]. Notably, GDM has been strongly associated with this predominant late-onset phenotype, which corresponds to the metabolic profile observed in GDM-related placental dysfunction [17]. Women with GDM frequently exhibit metabolic disturbances including hyperglycemia, dyslipidemia, and insulin resistance, which may collectively impair vascular adaptation during late gestation [18]. Persistent atherogenic dyslipidemia may contribute to endothelial dysfunction and increased vascular resistance, thereby increasing susceptibility to HDPs [19].

Physiological hyperlipidemia is a normal metabolic adaptation during pregnancy, with TG concentrations gradually increasing to support fetal growth and energy supply [20]. However, excessive elevation of TG may reflect pathological lipid dysregulation [21]. AIP may contribute to vascular dysfunction through several mechanisms. Elevated TG levels may promote lipid accumulation and oxidative stress [22–24], while reduced HDL-C may impair endothelial nitric oxide production and anti-inflammatory capacity [25–28]. These changes can lead to endothelial dysfunction, increased vascular resistance, and impaired placental perfusion, all of which are central features in the development of HDPs. Therefore, AIP may serve as an integrated indicator linking metabolic dysregulation with vascular pathology during pregnancy.

Our findings also highlight the potential value of cumulative lipid exposure. Pregnancy is characterized by dynamic metabolic changes, and single-time-point lipid measurements may not fully capture sustained metabolic stress [8]. By integrating repeated measurements, cumulative AIP may reflect long-term exposure to atherogenic dyslipidemia throughout pregnancy. This approach may help distinguish physiological gestational lipid changes from persistent metabolic abnormalities that contribute to vascular dysfunction. In addition, maternal metabolic disturbances may influence the intrauterine environment and fetal development [29, 30]. The observed linear dose–response relationship between AIP levels and the risk of NICU admission further suggests that maternal cardiometabolic disturbances may influence the intrauterine environment and neonatal outcomes.

Subgroup analyses showed that the associations between AIP and adverse outcomes were generally consistent across maternal age, pre-pregnancy BMI, and parity groups. This consistency suggests that lipid dysregulation may represent a shared metabolic pathway contributing to hypertensive complications in GDM, independent of traditional maternal risk factors. Validation analyses further confirmed the stability of these associations.

From a clinical perspective, AIP represents a simple and accessible biomarker derived from routine lipid testing. Incorporating AIP into prenatal metabolic assessment may improve risk stratification among women with GDM by providing complementary information beyond conventional metabolic indicators. Repeated evaluation of AIP during pregnancy may provide additional information compared with single measurements and help identify individuals at increased risk for HDPs. These findings support the potential role of dynamic lipid monitoring as part of a broader cardiometabolic assessment during pregnancy.

Several strengths of this study should be acknowledged. First, relatively few studies have examined the relationship between AIP and HDPs specifically in women with GDM. Second, we evaluated both single-time-point and cumulative AIP exposure, allowing us to assess dynamic metabolic changes during pregnancy. Third, the analysis adjusted for multiple potential confounders and included validation cohorts, which enhanced the robustness of the findings.

This study also has several limitations. First, detailed information on lifestyle factors, such as dietary habits and physical activity, was not available and may influence lipid metabolism. Second, lipid measurements were available only from the second and early third trimesters, and data from early pregnancy were lacking. Although gestational TG levels physiologically increase with advancing pregnancy [20], the cumulative AIP model inherently accounts for this dynamic trend, potentially enhancing its specificity for pathological dyslipidemia. In addition, there are no direct measurements of insulin resistance in our dataset, which may have limited the predictive performance of the clinical models. Future prospective studies should incorporate direct insulin resistance indices to improve risk stratification. In addition, integrative or weighted modeling strategies may be useful in future studies to clarify the relative contribution of different metabolic markers. Finally, the retrospective design limits causal inference. Future prospective studies with larger populations and serial lipid measurements throughout pregnancy are warranted to clarify the role of atherogenic lipid exposure in the development of hypertensive complications and long-term maternal and offspring metabolic health.

### Perspective of Asia

Gestational diabetes mellitus and hypertensive disorders of pregnancy are increasingly important maternal health problems in Asia. Given the high prevalence of metabolic abnormalities in Asian women, including dyslipidemia at relatively low BMI levels [31], practical biomarkers for early risk stratification are needed. In this study, both AIP and cumulative AIP were independently associated with preeclampsia, preeclampsia with severe features, and NICU admission in women with GDM. These findings suggest that lipid-derived markers may complement conventional clinical assessment in Asian obstetric practice. Further prospective studies in diverse Asian populations are needed to confirm their clinical utility and applicability across different healthcare settings.

## Conclusion

Elevated second-trimester and cumulative AIP levels were associated with increased risks of HDPs and neonatal complications in women with GDM. AIP may serve as a practical cardiometabolic marker reflecting the interaction between dyslipidemia and vascular dysfunction during pregnancy. Dynamic assessment of AIP may therefore help refine risk stratification for hypertensive complications in women with GDM.

## Supplementary information

Supplementary information
