# Supplementary material for: Atherogenic index of plasma and risk of hypertensive disorders of pregnancy in women with gestational diabetes mellitus: a two-center cohort study
Source: Hypertens Res. 2026 May 19;49(7):2086–96. doi: 10.1038/s41440-026-02684-8 (PMC13333490; doi:10.1038/s41440-026-02684-8)
Supplement: Supplementary file 1 — Supplementary information [file 41440_2026_2684_MOESM1_ESM.docx]

**Atherogenic index of plasma and risk of hypertensive disorders of pregnancy in women with gestational diabetes mellitus: a two-center cohort study**

**Supplementary information**

**Contents**

**Supplementary Tables**

Supplementary Table 1. Characteristics of the training and validation cohorts.

Supplementary Table 2. Maternal and neonatal outcomes according to AIP quartiles.

Supplementary Table 3. Maternal and neonatal outcomes according to cumulative AIP quartiles.

Supplementary Table 4. Hosmer–Lemeshow test of AIP for the training and validation cohorts.

Supplementary Table 5. Hosmer–Lemeshow test of cumulative AIP for the training cohort and validation cohorts.

Supplementary Table 6. Receiver operating characteristic curve analysis of AIP for detecting GDM-related maternal and neonatal outcomes.

Supplementary Table 7. Receiver operating characteristic curve analysis of cumulative AIP for detecting GDM-related maternal and neonatal outcomes.

Supplementary Table 8. Characteristics of the study population according to AIP quartiles in Validation cohort 1.

Supplementary Table 9. Characteristics of the study population according to cumulative AIP quartiles in Validation cohort 1.

Supplementary Table 10. Associations of AIP with maternal and neonatal outcomes in Validation cohort 1.

Supplementary Table 11. Associations of cumulative AIP with maternal and neonatal outcomes in Validation cohort 1.

Supplementary Table 12. Receiver operating characteristic curve analysis of AIP for detecting GDM-related maternal and neonatal outcomes in Validation cohort 1.

Supplementary Table 13. Receiver operating characteristic curve analysis of cumulative AIP for detecting GDM-related maternal and neonatal outcomes in Validation cohort 1.

Supplementary Table 14. Characteristics of the study population according to AIP quartiles in Validation cohort 2.

Supplementary Table 15. Characteristics of the study population according to cumulative AIP quartiles in Validation cohort 2.

Supplementary Table 16. Associations of AIP with maternal and neonatal outcomes in Validation cohort 2.

Supplementary Table 17. Associations of cumulative AIP with maternal and neonatal outcomes in Validation cohort 2.

Supplementary Table 18. Receiver operating characteristic curve analysis of AIP for detecting GDM-related maternal and neonatal outcomes in Validation cohort 2.

Supplementary Table 19. Receiver operating characteristic curve analysis of cumulative AIP for detecting GDM-related maternal and neonatal outcomes in Validation cohort 2.

**Supplementary Figures**

Supplementary Fig. 1. Multivariate RCS regression analysis for the nonlinear association of AIP and cumulative AIP with GDM-related maternal and neonatal outcomes.

Supplementary Fig. 2. Subgroup analysis for the association of AIP with GDM-related maternal and neonatal outcomes.

Supplementary Fig. 3. Subgroup analysis for the association of cumulative AIP with GDM-related maternal and neonatal outcomes.

Supplementary Table 1. Characteristics of the training and validation cohorts.

|  | Training cohort | Validation cohort 1 | Validation cohort 2 |
| --- | --- | --- | --- |
| **Maternal characteristics** |  |  |  |
| Age, years (SD) | 30.57 (4.04) | 30.60 (4.08) | 32.01 (4.53) |
| Pre-pregnancy BMI, kg/m^2^ (SD) | 23.23 (2.23) | 23.19 (2.15) | 23.88 (3.36) |
| Parity, *n* (%) |  |  |  |
| 1 | 1402 (65.54%) | 607 (66.27%) | 498 (54.61%) |
| ≥ 2 | 737 (34.46%) | 309 (33.73%) | 414 (45.39%) |
| OGTT-FPG, mmol/L (SD) | 4.78 (0.40) | 4.77 (0.42) | 4.92 (0.59) |
| OGTT-1 h, mmol/L (SD) | 8.93 (1.65) | 8.92 (1.68) | 9.68 (1.60) |
| OGTT-2 h, mmol/L (SD) | 7.78 (1.49) | 7.87 (1.47) | 8.43 (1.38) |
| HbA1c, % (SD) | 5.05 (0.32) | 5.05 (0.31) | 5.15 (0.40) |
| HbA1c, mmol/mol (SD) | 31.72 (3.47) | 31.67 (3.35) | 32.79 (4.36) |
| Second-trimester TC, mmol/L (SD) | 5.83 (0.95) | 5.85 (0.96) | 5.73 (0.97) |
| Second-trimester LDL-C, mmol/L (SD) | 3.00 (0.73) | 3.05 (0.70) | 2.92 (0.66) |
| **Neonatal characteristics** |  |  |  |
| Gestational age, weeks (SD) | 39.35 (1.22) | 39.39 (1.22) | 39.01 (1.18) |
| Birth weight, g (SD) | 3407.83 (431.37) | 3418.43 (431.33) | 3371.24 (464.04) |

**Note:** Data are presented as mean (standard deviation) or *n* (%). The bold *P* value indicates statistical significance.

**Abbreviations:** BMI, body mass index; FPG, fasting plasma glucose; HbA1c, glycated hemoglobin; LDL-C, low-density lipoprotein-cholesterol; OGTT, oral glucose tolerance test; Q, quartiles; TC, total cholesterol.

Supplementary Table 2. Maternal and neonatal outcomes according to AIP quartiles.

|  | Overall (*n* = 2,139) | Q1 (*n* = 535) | Q2 (*n* = 535) | Q3 (*n* = 535) | Q4 (*n* = 534) | *P* for trend |
| --- | --- | --- | --- | --- | --- | --- |
| **Maternal outcomes** |  |  |  |  |  |  |
| PE | 43 (2.01%) | 2 (0.37%) | 8 (1.50%) | 13 (2.43%) | 20 (3.75%) | **<0.001** |
| PE with severe features | 16 (0.75%) | 0 (0.00%) | 0 (0.00%) | 5 (0.93%) | 11 (2.06%) | **<0.001** |
| Oligohydramnios | 42 (1.96%) | 10 (1.87%) | 7 (1.31%) | 14 (2.62%) | 11 (2.06%) | 0.483 |
| Polyhydramnios | 57 (2.66%) | 13 (2.43%) | 19 (3.55%) | 15 (2.80%) | 10 (1.87%) | 0.438 |
| ICP | 20 (0.94%) | 8 (1.50%) | 5 (0.93%) | 2 (0.37%) | 5 (0.94%) | 0.229 |
| Fetal distress | 133 (6.22%) | 28 (5.23%) | 32 (5.98%) | 34 (6.36%) | 39 (7.30%) | 0.159 |
| Postpartum hemorrhage | 149 (6.97%) | 33 (6.17%) | 39 (7.29%) | 37 (6.92%) | 40 (7.49%) | 0.466 |
| **Neonatal outcomes** |  |  |  |  |  |  |
| TTN | 4 (0.19%) | 1 (0.19%) | 0 (0.00%) | 1 (0.19%) | 2 (0.37%) | 0.370 |
| RDS | 4 (0.19%) | 0 (0.00%) | 1 (0.19%) | 3 (0.56%) | 0 (0.00%) | 0.653 |
| Asphyxia of newborn | 2 (0.09%) | 0 (0.00%) | 0 (0.00%) | 0 (0.00%) | 2 (0.37%) | 0.713 |
| Neonatal hypoglycemia | 11 (0.51%) | 3 (0.56%) | 0 (0.00%) | 4 (0.75%) | 4 (0.75%) | 0.343 |
| Neonatal hyperbilirubinemia | 25 (1.17%) | 5 (0.93%) | 9 (1.68%) | 6 (1.12%) | 5 (0.94%) | 0.790 |
| LGA | 190 (8.88%) | 48 (8.97%) | 42 (7.85%) | 46 (8.60%) | 54 (10.11%) | 0.449 |
| SGA | 63 (2.95%) | 8 (1.50%) | 23 (4.30%) | 20 (3.74%) | 12 (2.25%) | 0.603 |
| Admission to NICU | 75 (3.51%) | 12 (2.24%) | 15 (2.80%) | 20 (3.74%) | 28 (5.24%) | **0.005** |

**Note:** Data are presented as *n* (%). The bold *P* value indicates statistical significance.

**Abbreviations:** AIP, atherogenic index of plasma; GDM, gestational diabetes mellitus; ICP, intrahepatic cholestasis of pregnancy; LGA, large for gestational age; NICU, neonatal intensive care unit; PE, preeclampsia; Q, quartiles; RDS, respiratory distress syndrome; SGA, small for gestational age; TTN, transient tachypnea of the newborn.

Supplementary Table 3. Maternal and neonatal outcomes according to cumulative AIP quartiles.

|  | Overall (*n* = 2,139) | Q1 (*n* = 535) | Q2 (*n* = 535) | Q3 (*n* = 535) | Q4 (*n* = 534) | *P* for trend |
| --- | --- | --- | --- | --- | --- | --- |
| **Maternal outcomes** |  |  |  |  |  |  |
| PE | 43 (2.01%) | 2 (0.37%) | 7 (1.31%) | 13 (2.43%) | 21 (3.93%) | **<0.001** |
| PE with severe features | 16 (0.75%) | 0 (0.00%) | 1 (0.19%) | 2 (0.37%) | 13 (2.43%) | **<0.001** |
| Oligohydramnios | 42 (1.96%) | 10 (1.87%) | 6 (1.12%) | 15 (2.80%) | 11 (2.06%) | 0.401 |
| Polyhydramnios | 57 (2.66%) | 13 (2.43%) | 22 (4.11%) | 12 (2.24%) | 10 (1.87%) | 0.256 |
| ICP | 20 (0.94%) | 5 (0.93%) | 7 (1.31%) | 5 (0.93%) | 3 (0.56%) | 0.423 |
| Fetal distress | 133 (6.22%) | 34 (6.36%) | 27 (5.05%) | 34 (6.36%) | 38 (7.12%) | 0.442 |
| Postpartum hemorrhage | 149 (6.97%) | 29 (5.42%) | 41 (7.66%) | 37 (6.92%) | 42 (7.87%) | 0.181 |
| **Neonatal outcomes** |  |  |  |  |  |  |
| TTN | 4 (0.19%) | 1 (0.19%) | 0 (0.00%) | 1 (0.19%) | 2 (0.37%) | 0.370 |
| RDS | 4 (0.19%) | 0 (0.00%) | 2 (0.37%) | 1 (0.19%) | 1 (0.19%) | 0.653 |
| Asphyxia of newborn | 2 (0.09%) | 1 (0.19%) | 3 (0.56%) | 1 (0.19%) | 1 (0.19%) | 0.716 |
| Neonatal hypoglycemia | 11 (0.51%) | 3 (0.56%) | 1 (0.19%) | 3 (0.56%) | 4 (0.75%) | 0.498 |
| Neonatal hyperbilirubinemia | 25 (1.17%) | 3 (0.56%) | 11 (2.06%) | 6 (1.12%) | 5 (0.94%) | 0.926 |
| LGA | 190 (8.88%) | 40 (7.48%) | 47 (8.79%) | 47 (8.79%) | 56 (10.49%) | 0.101 |
| SGA | 63 (2.95%) | 11 (2.06%) | 20 (3.74%) | 20 (3.74%) | 12 (2.25%) | 0.860 |
| Admission to NICU | 75 (3.51%) | 11 (2.06%) | 17 (3.18%) | 21 (3.93%) | 26 (4.87%) | **0.010** |

**Note:** Data are presented as *n* (%). The bold *P* value indicates statistical significance.

**Abbreviations:** AIP, atherogenic index of plasma; GDM, gestational diabetes mellitus; ICP, intrahepatic cholestasis of pregnancy; LGA, large for gestational age; NICU, neonatal intensive care unit; PE, preeclampsia; Q, quartiles; RDS, respiratory distress syndrome; SGA, small for gestational age; TTN, transient tachypnea of the newborn.

Supplementary Table 4. Hosmer–Lemeshow test of AIP for the training and validation cohorts.

| Outcome | Model | Training cohort | | Validation cohort 1 | | Validation cohort 2 | |
| --- | --- | --- | --- | --- | --- | --- | --- |
|  |  | χ^2^ | *P* value | χ^2^ | *P* value | χ^2^ | *P* value |
| PE | 1 | <0.001 | >0.999 | 13.848 | 0.086 | 7.377 | 0.497 |
|  | 2 | 7.241 | 0.511 | 10.889 | 0.208 | 6.736 | 0.565 |
|  | 3 | 7.149 | 0.521 | 8.243 | 0.410 | 4.526 | 0.807 |
| PE with severe features | 1 | 6.524 | 0.589 | 9.509 | 0.301 | 5.513 | 0.702 |
|  | 2 | 7.978 | 0.436 | 11.118 | 0.195 | 6.112 | 0.635 |
|  | 3 | 8.184 | 0.416 | 4.884 | 0.770 | 3.267 | 0.917 |
| NICU | 1 | 5.172 | 0.739 | 8.088 | 0.425 | 6.593 | 0.581 |
|  | 2 | 3.913 | 0.865 | 3.568 | 0.894 | 8.448 | 0.391 |
|  | 3 | 2.538 | 0.960 | 4.026 | 0.855 | 6.593 | 0.581 |

**Note:** Model 1 was unadjusted. Model 2 was Model 1 adjusted for age, pre-pregnancy BMI, and parity. Model 3 was Model 2 adjusted for TC, LDL-C, OGTT-FPG, and HbA1c. The bold *P* value indicates statistical significance.

**Abbreviations:** NICU, neonatal intensive care unit; PE, preeclampsia.

Supplementary Table 5. Hosmer–Lemeshow test of cumulative AIP for the training cohort and validation cohorts.

| Outcome | Model | Training cohort | | Validation cohort 1 | | Validation cohort 2 | |
| --- | --- | --- | --- | --- | --- | --- | --- |
|  |  | χ^2^ | *P* value | χ^2^ | *P* value | χ^2^ | *P* value |
| PE | 1 | 8.876 | 0.353 | 4.839 | 0.775 | 4.991 | 0.759 |
|  | 2 | 8.950 | 0.347 | 9.473 | 0.304 | 6.778 | 0.561 |
|  | 3 | 3.299 | 0.914 | 10.903 | 0.207 | 16.800 | 0.052 |
| PE with severe features | 1 | 11.858 | 0.158 | 4.449 | 0.815 | 3.127 | 0.926 |
|  | 2 | 11.664 | 0.167 | 7.689 | 0.465 | 2.699 | 0.952 |
|  | 3 | 3.925 | 0.864 | 7.737 | 0.460 | 7.174 | 0.518 |
| NICU | 1 | 8.899 | 0.351 | 11.205 | 0.190 | 5.029 | 0.754 |
|  | 2 | 9.784 | 0.281 | 3.236 | 0.919 | 2.569 | 0.958 |
|  | 3 | 8.174 | 0.226 | 7.388 | 0.496 | 5.787 | 0.671 |

**Note:** Model 1 was unadjusted. Model 2 was Model 1 adjusted for age, pre-pregnancy BMI, and parity. Model 3 was Model 2 adjusted for TC, LDL-C, OGTT-FPG, and HbA1c. The bold *P* value indicates statistical significance.

**Abbreviations:** NICU, neonatal intensive care unit; PE, preeclampsia.

Supplementary Table 6. Receiver operating characteristic curve analysis of AIP for detecting GDM-related maternal and neonatal outcomes.

|  | Variables | AUC (95% CI) | Best threshold | Specificity | Sensitivity | Youden index | *P* value |
| --- | --- | --- | --- | --- | --- | --- | --- |
| PE | AIP | 0.692 (0.617–0.767) | -0.134 | 0.459 | 0.860 | 0.320 |  |
|  | Clinical variables | 0.699 (0.616–0.783) | 0.021 | 0.714 | 0.605 | 0.318 |  |
|  | Combined | 0.780 (0.714–0.847) | 0.017 | 0.653 | 0.860 | 0.513 | **0.025** |
| PE with severe features | AIP | 0.823 (0.744–0.902) | 0.190 | 0.597 | 0.938 | 0.535 |  |
|  | Clinical variables | 0.729 (0.611–0.848) | 0.007 | 0.632 | 0.750 | 0.382 |  |
|  | Combined | 0.874 (0.808–0.940) | 0.007 | 0.793 | 0.875 | 0.668 | **0.002** |
| Admission to NICU | AIP | 0.593 (0.525–0.662) | 0.299 | 0.634 | 0.533 | 0.167 |  |
|  | Clinical variables | 0.588 (0.523–0.653) | 0.040 | 0.710 | 0.440 | 0.150 |  |
|  | Combined | 0.643 (0.578–0.709) | 0.040 | 0.732 | 0.547 | 0.278 | **0.029** |

**Note:** Clinical variables included age, pre-pregnancy BMI, parity, TC, LDL-C, OGTT-FPG, and HbA1c. The *P* value indicates differences between clinical variables AUC and AIP + clinical variables (combined) AUC. The bold *P* value indicates statistical significance.

**Abbreviations:** AIP, atherogenic index of plasma; AUC, area under the curve; GDM, gestational diabetes mellitus; NICU, neonatal intensive care unit; PE, preeclampsia.

Supplementary Table 7. Receiver operating characteristic curve analysis of cumulative AIP for detecting GDM-related maternal and neonatal outcomes.

|  | Variables | AUC (95% CI) | Best threshold | Specificity | Sensitivity | Youden index | *P* value |
| --- | --- | --- | --- | --- | --- | --- | --- |
| PE | Cumulative AIP | 0.702 (0.629–0.775) | 0.025 | 0.531 | 0.791 | 0.322 |  |
|  | Clinical variables | 0.699 (0.616–0.783) | 0.021 | 0.714 | 0.605 | 0.318 |  |
|  | Combined | 0.781 (0.714–0.849) | 0.015 | 0.607 | 0.860 | 0.467 | **0.016** |
| PE with severe features | Cumulative AIP | 0.852 (0.783–0.920) | 0.504 | 0.707 | 0.938 | 0.644 |  |
|  | Clinical variables | 0.729 (0.611–0.848) | 0.007 | 0.632 | 0.750 | 0.382 |  |
|  | Combined | 0.885 (0.814–0.957) | 0.006 | 0.785 | 0.875 | 0.660 | **<0.001** |
| Admission to NICU | Cumulative AIP | 0.580 (0.513–0.647) | -0.199 | 0.435 | 0.720 | 0.155 |  |
|  | Clinical variables | 0.588 (0.523–0.653) | 0.040 | 0.710 | 0.440 | 0.150 |  |
|  | Combined | 0.634 (0.570–0.698) | 0.038 | 0.671 | 0.587 | 0.257 | **0.033** |

**Note:** Clinical variables included age, pre-pregnancy BMI, parity, TC, LDL-C, OGTT-FPG, and HbA1c. The *P* value indicates differences between clinical variables AUC and AIP + clinical variables (combined) AUC. The bold *P* value indicates statistical significance.

**Abbreviations:** AIP, atherogenic index of plasma; AUC, area under the curve; GDM, gestational diabetes mellitus; NICU, neonatal intensive care unit; PE, preeclampsia.

Supplementary Table 8. Characteristics of the study population according to AIP quartiles in Validation cohort 1.

| Characteristics | AIP quartiles | | | | *P* value |
| --- | --- | --- | --- | --- | --- |
|  | Q1 (*n* = 229) | Q2 (*n* = 229) | Q3 (*n* = 229) | Q4 (*n* = 229) |  |
| **Maternal characteristics** |  |  |  |  |  |
| Age, years (SD) | 30.14 (3.86) | 30.54 (4.16) | 30.80 (4.34) | 30.93 (3.92) | 0.167 |
| Pre-pregnancy BMI, kg/m^2^ (SD) | 22.79 (1.84) | 23.09 (2.32) | 23.23 (2.30) | 23.66 (2.03) | **<0.001** |
| Parity, *n* (%) |  |  |  |  | 0.126 |
| 1 | 162 (70.74%) | 141 (61.57%) | 158 (69.00%) | 146 (63.76%) |  |
| ≥ 2 | 67 (29.26%) | 88 (38.43%) | 71 (31.00%) | 83 (36.24%) |  |
| OGTT-FPG, mmol/L (SD) | 4.72 (0.45) | 4.74 (0.39) | 4.81 (0.43) | 4.84 (0.41) | **0.007** |
| OGTT-1 h, mmol/L (SD) | 8.80 (1.75) | 8.93 (1.59) | 8.95 (1.63) | 9.02 (1.77) | 0.536 |
| OGTT-2 h, mmol/L (SD) | 7.79 (1.53) | 7.85 (1.41) | 7.90 (1.46) | 7.92 (1.48) | 0.771 |
| HbA1c, % (SD) | 4.99 (0.27) | 5.05 (0.32) | 5.06 (0.32) | 5.09 (0.30) | **0.003** |
| HbA1c, mmol/mol (SD) | 31.03 (2.90) | 31.67 (3.54) | 31.83 (3.51) | 32.16 (3.31) | **0.003** |
| Second-trimester TC, mmol/L (SD) | 5.98 (0.95) | 5.99 (0.89) | 5.86 (0.99) | 5.60 (0.96) | **<0.001** |
| Second-trimester LDL-C, mmol/L (SD) | 3.10 (0.67) | 3.07 (0.66) | 3.12 (0.74) | 2.93 (0.74) | **0.015** |
| **Neonatal characteristics** |  |  |  |  |  |
| Gestational age, weeks (SD) | 39.50 (1.16) | 39.42 (1.14) | 39.30 (1.29) | 39.34 (1.27) | 0.294 |
| Birth weight, g (SD) | 3372.27 (401.62) | 3435.50 (415.85) | 3386.38 (446.75) | 3479.56 (453.43) | **0.031** |

**Note:** Data are presented as mean (standard deviation) or *n* (%). The bold *P* value indicates statistical significance.

**Abbreviations:** AIP, atherogenic index of plasma; BMI, body mass index; FPG, fasting plasma glucose; HbA1c, glycated hemoglobin; LDL-C, low-density lipoprotein-cholesterol; OGTT, oral glucose tolerance test; Q, quartiles; TC, total cholesterol.

Supplementary Table 9. Characteristics of the study population according to cumulative AIP quartiles in Validation cohort 1.

| Characteristics | Cumulative AIP quartiles | | | | *P* value |
| --- | --- | --- | --- | --- | --- |
|  | Q1 (*n* = 229) | Q2 (*n* = 229) | Q3 (*n* = 229) | Q4 (*n* = 229) |  |
| **Maternal characteristics** |  |  |  |  |  |
| Age, years (SD) | 30.35 (3.92) | 30.22 (4.13) | 30.69 (4.12) | 31.16 (4.11) | 0.063 |
| Pre-pregnancy BMI, kg/m^2^ (SD) | 22.88 (2.02) | 22.92 (2.12) | 23.37 (2.44) | 23.60 (1.92) | **<0.001** |
| Parity, *n* (%) |  |  |  |  | 0.860 |
| 1 | 157 (68.56%) | 150 (65.50%) | 151 (65.94%) | 149 (65.07%) |  |
| ≥ 2 | 72 (31.44%) | 79 (34.50%) | 78 (34.06%) | 80 (34.93%) |  |
| OGTT-FPG, mmol/L (SD) | 4.72 (0.46) | 4.76 (0.44) | 4.77 (0.36) | 4.85 (0.43) | **0.011** |
| OGTT-1 h, mmol/L (SD) | 8.77 (1.72) | 8.86 (1.65) | 9.02 (1.61) | 9.05 (1.75) | 0.217 |
| OGTT-2 h, mmol/L (SD) | 7.78 (1.48) | 7.79 (1.52) | 7.89 (1.46) | 8.00 (1.42) | 0.341 |
| HbA1c, % (SD) | 5.01 (0.27) | 5.02 (0.32) | 5.06 (0.31) | 5.10 (0.32) | **0.002** |
| HbA1c, mmol/mol (SD) | 31.23 (2.97) | 31.35 (3.46) | 31.83 (3.36) | 32.29 (3.48) | **0.002** |
| Second-trimester TC, mmol/L (SD) | 5.91 (0.89) | 6.02 (1.00) | 5.87 (0.95) | 5.62 (0.95) | **<0.001** |
| Second-trimester LDL-C, mmol/L (SD) | 3.04 (0.63) | 3.12 (0.73) | 3.12 (0.72) | 2.94 (0.73) | **0.015** |
| **Neonatal characteristics** |  |  |  |  |  |
| Gestational age, weeks (SD) | 39.52 (1.12) | 39.39 (1.25) | 39.36 (1.12) | 39.28 (1.37) | 0.228 |
| Birth weight, g (SD) | 3369.17 (396.17) | 3401.40 (444.42) | 3411.14 (396.28) | 3492.01 (476.35) | **0.018** |

**Note:** Data are presented as mean (standard deviation) or *n* (%). The bold *P* value indicates statistical significance.

**Abbreviations:** AIP, atherogenic index of plasma; BMI, body mass index; FPG, fasting plasma glucose; HbA1c, glycated hemoglobin; LDL-C, low-density lipoprotein-cholesterol; OGTT, oral glucose tolerance test; Q, quartiles; TC, total cholesterol.

Supplementary Table 10. Associations of AIP with maternal and neonatal outcomes in Validation cohort 1.

|  | Continuous | AIP quartiles | | | | *P* for trend |
| --- | --- | --- | --- | --- | --- | --- |
|  |  | Q1 | Q2 | Q3 | Q4 |  |
| PE |  |  |  |  |  |  |
| Model 1 | 2.14 (1.54–3.00) | Ref. | 3.05 (0.70–20.99) | 4.11 (1.02–27.42) | 9.10 (2.57–57.84) | **<0.001** |
| Model 2 | 2.05 (1.45–2.90) | Ref. | 2.72 (0.60–18.94) | 3.41 (0.83–22.95) | 7.53 (2.09–48.20) | **0.001** |
| Model 3 | 2.03 (1.42–2.92) | Ref. | 2.55 (0.56–17.80) | 3.13 (0.75–21.20) | 6.87 (1.88–44.25) | **0.003** |
| PE with severe features |  |  |  |  |  |  |
| Model 1 | 2.51 (1.61–3.95) | Ref. | 3.03 (0.38–61.43) | 3.03 (0.38–61.43) | 10.41 (1.97–191.87) | **0.006** |
| Model 2 | 2.38 (1.51–3.79) | Ref. | 2.55 (0.32–52.25) | 2.61 (0.33–53.29) | 8.38 (1.55–155.55) | **0.011** |
| Model 3 | 2.42 (1.50–3.96) | Ref. | 2.27 (0.28–46.61) | 2.27 (0.28–47.18) | 7.35 (1.31–138.73) | **0.018** |
| Admission to NICU |  |  |  |  |  |  |
| Model 1 | 1.84 (1.32–2.57) | Ref. | 1.26 (0.33–5.13) | 2.30 (0.74–8.59) | 3.94 (1.41–14.00) | **0.004** |
| Model 2 | 1.88 (1.34–2.66) | Ref. | 1.30 (0.34–5.35) | 2.19 (0.69–8.23) | 3.98 (1.40–14.31) | **0.006** |
| Model 3 | 1.97 (1.38–2.82) | Ref. | 1.33 (0.34–5.47) | 2.35 (0.73–8.95) | 4.38 (1.50–16.09) | **0.004** |

**Note:** Model 1 was unadjusted. Model 2 was Model 1 adjusted for age, pre-pregnancy BMI, and parity. Model 3 was Model 2 adjusted for TC, LDL-C, OGTT-FPG, and HbA1c. The bold *P* value indicates statistical significance.

**Abbreviations:** AIP, atherogenic index of plasma; GDM, gestational diabetes mellitus; NICU, neonatal intensive care unit; PE, preeclampsia; Q, quartiles.

Supplementary Table 11. Associations of cumulative AIP with maternal and neonatal outcomes in Validation cohort 1.

|  | Continuous | Cumulative AIP quartiles | | | | *P* for trend |
| --- | --- | --- | --- | --- | --- | --- |
|  |  | Q1 | Q2 | Q3 | Q4 |  |
| PE |  |  |  |  |  |  |
| Model 1 | 2.10 (1.52–2.92) | Ref. | 1.51 (0.25–11.52) | 5.73 (1.52–37.29) | 9.10 (2.57–57.84) | **<0.001** |
| Model 2 | 2.00 (1.44–2.81) | Ref. | 1.58 (0.26–12.16) | 5.02 (1.30–33.01) | 8.18 (2.28–52.39) | **<0.001** |
| Model 3 | 1.93 (1.38–2.74) | Ref. | 1.51 (0.24–11.62) | 4.87 (1.26–32.11) | 7.49 (2.06–48.26) | **0.001** |
| PE with severe features * |  |  |  |  |  |  |
| Model 1 | 2.45 (1.60–3.80) | Ref. | 0.33 (0.01–6.25) | 4.43 (0.92–42.75) | 7.29 (1.69–67.92) | **<0.001** |
| Model 2 | 2.37 (1.54–3.71) | Ref. | 0.32 (0.01–6.11) | 3.82 (0.78–36.98) | 6.43 (1.49–59.99) | **<0.001** |
| Model 3 | 2.27 (1.45–3.60) | Ref. | 0.30 (0.01–5.70) | 3.67 (0.74–36.04) | 5.69 (1.26–54.34) | **0.001** |
| Admission to NICU |  |  |  |  |  |  |
| Model 1 | 1.84 (1.33–2.54) | Ref. | 2.38 (0.65–11.13) | 3.44 (1.04–15.49) | 4.53 (1.44–19.97) | **0.011** |
| Model 2 | 1.85 (1.33–2.59) | Ref. | 2.48 (0.68–11.62) | 3.42 (1.02–15.48) | 4.43 (1.39–19.64) | **0.015** |
| Model 3 | 1.93 (1.37–2.73) | Ref. | 2.49 (0.68–11.74) | 3.63 (1.07–16.57) | 4.72 (1.44–21.30) | **0.012** |

**Note:** Model 1 was unadjusted. Model 2 was Model 1 adjusted for age, pre-pregnancy BMI, and parity. Model 3 was Model 2 adjusted for TC, LDL-C, OGTT-FPG, and HbA1c. The bold *P* value indicates statistical significance. Firth's bias-reduced logistic regression was applied due to complete separation in the first quartile group of PE with severe features.

**Abbreviations:** AIP, atherogenic index of plasma; GDM, gestational diabetes mellitus; NICU, neonatal intensive care unit; PE, preeclampsia; Q, quartiles.

Supplementary Table 12. Receiver operating characteristic curve analysis of AIP for detecting GDM-related maternal and neonatal outcomes in Validation cohort 1.

|  | Variables | AUC (95% CI) | Best threshold | Specificity | Sensitivity | Youden index | *P* value |
| --- | --- | --- | --- | --- | --- | --- | --- |
| PE | AIP | 0.726 (0.642–0.810) | 1.082 | 0.862 | 0.515 | 0.377 |  |
|  | Clinical variables | 0.724 (0.640–0.808) | 0.038 | 0.710 | 0.667 | 0.377 |  |
|  | Combined | 0.800 (0.740–0.859) | 0.029 | 0.626 | 0.879 | 0.505 | **0.029** |
| PE with severe features | AIP | 0.761 (0.640–0.883) | 1.181 | 0.882 | 0.588 | 0.470 |  |
|  | Clinical variables | 0.703 (0.560–0.846) | 0.031 | 0.863 | 0.529 | 0.393 |  |
|  | Combined | 0.834 (0.756–0.911) | 0.015 | 0.684 | 0.882 | 0.566 | **0.023** |
| Admission to NICU | AIP | 0.662 (0.564–0.759) | 0.522 | 0.725 | 0.606 | 0.331 |  |
|  | Clinical variables | 0.582 (0.475–0.690) | 0.042 | 0.750 | 0.455 | 0.204 |  |
|  | Combined | 0.702 (0.606–0.798) | 0.036 | 0.665 | 0.667 | 0.331 | **0.040** |

**Note:** Clinical variables included age, pre-pregnancy BMI, parity, TC, LDL-C, OGTT-FPG, and HbA1c. The *P* value indicates differences between clinical variables AUC and AIP + clinical variables (combined) AUC. The bold *P* value indicates statistical significance.

**Abbreviations:** AIP, atherogenic index of plasma; AUC, area under the curve; GDM, gestational diabetes mellitus; NICU, neonatal intensive care unit; PE, preeclampsia.

Supplementary Table 13. Receiver operating characteristic curve analysis of cumulative AIP for detecting GDM-related maternal and neonatal outcomes in Validation cohort 1.

|  | Variables | AUC (95% CI) | Best threshold | Specificity | Sensitivity | Youden index | *P* value |
| --- | --- | --- | --- | --- | --- | --- | --- |
| PE | Cumulative AIP | 0.741 (0.663–0.819) | -0.065 | 0.511 | 0.879 | 0.390 |  |
|  | Clinical variables | 0.724 (0.640–0.808) | 0.038 | 0.710 | 0.667 | 0.377 |  |
|  | Combined | 0.800 (0.740–0.861) | 0.024 | 0.574 | 0.909 | 0.483 | **0.016** |
| PE with severe features | Cumulative AIP | 0.797 (0.700–0.894) | 0.252 | 0.641 | 0.882 | 0.523 |  |
|  | Clinical variables | 0.703 (0.560–0.846) | 0.031 | 0.863 | 0.529 | 0.393 |  |
|  | Combined | 0.844 (0.776–0.913) | 0.018 | 0.736 | 0.882 | 0.619 | **0.006** |
| Admission to NICU | Cumulative AIP | 0.662 (0.572–0.753) | -0.280 | 0.412 | 0.879 | 0.291 |  |
|  | Clinical variables | 0.582 (0.475–0.690) | 0.042 | 0.750 | 0.455 | 0.204 |  |
|  | Combined | 0.695 (0.602–0.789) | 0.041 | 0.735 | 0.606 | 0.341 | **0.037** |

**Note:** Clinical variables included age, pre-pregnancy BMI, parity, TC, LDL-C, OGTT-FPG, and HbA1c. The *P* value indicates differences between clinical variables AUC and AIP + clinical variables (combined) AUC. The bold *P* value indicates statistical significance.

**Abbreviations:** AIP, atherogenic index of plasma; AUC, area under the curve; GDM, gestational diabetes mellitus; NICU, neonatal intensive care unit; PE, preeclampsia.

Supplementary Table 14. Characteristics of the study population according to AIP quartiles in Validation cohort 2.

| Characteristics | AIP quartiles | | | | *P* value |
| --- | --- | --- | --- | --- | --- |
|  | Q1 (*n* = 228) | Q2 (*n* = 228) | Q3 (*n* = 228) | Q4 (*n* = 228) |  |
| **Maternal characteristics** |  |  |  |  |  |
| Age, years (SD) | 31.98 (4.50) | 32.02 (4.41) | 32.16 (4.42) | 31.86 (4.81) | 0.921 |
| Pre-pregnancy BMI, kg/m^2^ (SD) | 23.25 (3.25) | 23.77 (3.43) | 24.20 (2.92) | 24.29 (3.69) | **0.003** |
| Parity, *n* (%) |  |  |  |  | 0.327 |
| 1 | 120 (52.63%) | 128 (56.14%) | 116 (50.88%) | 134 (58.77%) |  |
| ≥ 2 | 108 (47.37%) | 100 (43.86%) | 112 (49.12%) | 94 (41.23%) |  |
| OGTT-FPG, mmol/L (SD) | 4.75 (0.55) | 4.89 (0.54) | 4.97 (0.63) | 5.07 (0.60) | **<0.001** |
| OGTT-1 h, mmol/L (SD) | 9.54 (1.60) | 9.61 (1.59) | 9.61 (1.60) | 9.97 (1.58) | **0.018** |
| OGTT-2 h, mmol/L (SD) | 8.45 (1.24) | 8.38 (1.45) | 8.36 (1.42) | 8.55 (1.40) | 0.462 |
| HbA1c, % (SD) | 5.13 (0.40) | 5.12 (0.39) | 5.15 (0.41) | 5.20 (0.40) | 0.106 |
| HbA1c, mmol/mol (SD) | 32.19 (4.14) | 32.32 (4.21) | 32.59 (3.99) | 32.99 (4.10) | 0.106 |
| Second-trimester TC, mmol/L (SD) | 6.15 (1.03) | 5.83 (0.94) | 5.65 (0.88) | 5.30 (0.81) | **<0.001** |
| Second-trimester LDL-C, mmol/L (SD) | 3.05 (0.68) | 2.98 (0.68) | 2.96 (0.63) | 2.70 (0.61) | **<0.001** |
| **Neonatal characteristics** |  |  |  |  |  |
| Gestational age, weeks (SD) | 39.10 (1.08) | 39.00 (1.12) | 39.00 (1.17) | 38.95 (1.35) | 0.598 |
| Birth weight, g (SD) | 3331.89 (368.47) | 3316.32 (430.52) | 3375.31 (425.84) | 3461.45 (590.72) | **0.004** |

**Note:** Data are presented as mean (standard deviation) or *n* (%). The bold *P* value indicates statistical significance.

**Abbreviations:** AIP, atherogenic index of plasma; BMI, body mass index; FPG, fasting plasma glucose; HbA1c, glycated hemoglobin; LDL-C, low-density lipoprotein-cholesterol; OGTT, oral glucose tolerance test; Q, quartiles; TC, total cholesterol.

Supplementary Table 15. Characteristics of the study population according to cumulative AIP quartiles in Validation cohort 2.

| Characteristics | Cumulative AIP quartiles | | | | *P* value |
| --- | --- | --- | --- | --- | --- |
|  | Q1 (*n* = 228) | Q2 (*n* = 228) | Q3 (*n* = 228) | Q4 (*n* = 228) |  |
| **Maternal characteristics** |  |  |  |  |  |
| Age, years (SD) | 32.00 (4.66) | 32.02 (4.34) | 31.97 (4.46) | 32.03 (4.69) | 0.999 |
| Pre-pregnancy BMI, kg/m^2^ (SD) | 23.14 (3.19) | 23.62 (3.12) | 23.93 (3.27) | 24.81 (3.62) | **<0.001** |
| Parity, *n* (%) |  |  |  |  | 0.271 |
| 1 | 117 (51.32%) | 119 (52.19%) | 136 (59.65%) | 126 (55.26%) |  |
| ≥ 2 | 111 (48.68%) | 109 (47.81%) | 92 (40.35%) | 102 (44.74%) |  |
| OGTT-FPG, mmol/L (SD) | 4.69 (0.49) | 4.87 (0.57) | 4.94 (0.58) | 5.17 (0.61) | **<0.001** |
| OGTT-1 h, mmol/L (SD) | 9.46 (1.53) | 9.74 (1.56) | 9.71 (1.59) | 9.82 (1.70) | 0.103 |
| OGTT-2 h, mmol/L (SD) | 8.41 (1.26) | 8.40 (1.50) | 8.43 (1.34) | 8.49 (1.41) | 0.889 |
| HbA1c, % (SD) | 5.07 (0.38) | 5.15 (0.39) | 5.17 (0.39) | 5.21 (0.43) | **0.002** |
| HbA1c, mmol/mol (SD) | 31.84 (3.84) | 32.26 (4.08) | 32.93 (4.14) | 33.13 (4.30) | **0.002** |
| Second-trimester TC, mmol/L (SD) | 6.19 (1.05) | 5.73 (0.89) | 5.56 (0.87) | 5.45 (0.89) | **<0.001** |
| Second-trimester LDL-C, mmol/L (SD) | 3.12 (0.71) | 2.93 (0.64) | 2.86 (0.62) | 2.78 (0.63) | **<0.001** |
| **Neonatal characteristics** |  |  |  |  |  |
| Gestational age, weeks (SD) | 39.15 (0.94) | 38.98 (1.00) | 38.95 (1.39) | 38.96 (1.33) | 0.237 |
| Birth weight, g (SD) | 3329.25 (371.80) | 3324.91 (395.97) | 3347.19 (478.13) | 3483.60 (568.65) | **<0.001** |

**Note:** Data are presented as mean (standard deviation) or *n* (%). The bold *P* value indicates statistical significance.

**Abbreviations:** AIP, atherogenic index of plasma; BMI, body mass index; FPG, fasting plasma glucose; HbA1c, glycated hemoglobin; LDL-C, low-density lipoprotein-cholesterol; OGTT, oral glucose tolerance test; Q, quartiles; TC, total cholesterol.

Supplementary Table 16. Associations of AIP with maternal and neonatal outcomes in Validation cohort 2.

|  | Continuous | AIP quartiles | | | | *P* for trend |
| --- | --- | --- | --- | --- | --- | --- |
|  |  | Q1 | Q2 | Q3 | Q4 |  |
| PE |  |  |  |  |  |  |
| Model 1 | 2.09 (1.62–2.72) | Ref. | 1.41 (0.44–4.84) | 4.29 (1.70–13.07) | 5.74 (2.35–17.22) | **<0.001** |
| Model 2 | 1.87 (1.43–2.47) | Ref. | 1.22 (0.38–4.24) | 3.83 (1.50–11.79) | 4.54 (1.82–13.81) | **<0.001** |
| Model 3 | 1.92 (1.45–2.59) | Ref. | 1.34 (0.41–4.76) | 4.61 (1.70–14.91) | 5.18 (1.90–16.88) | **<0.001** |
| PE with severe features |  |  |  |  |  |  |
| Model 1 | 2.52 (1.67–3.84) | Ref. | 3.03 (0.38–61.44) | 4.05 (0.59–79.63) | 10.41 (1.97–191.91) | **0.005** |
| Model 2 | 2.09 (1.35–3.26) | Ref. | 2.63 (0.33–53.70) | 3.52 (0.51–69.35) | 7.53 (1.37–140.30) | **0.020** |
| Model 3 | 2.14 (1.34–3.46) | Ref. | 2.78 (0.34–57.78) | 3.83 (0.51–78.64) | 7.43 (1.19–146.51) | **0.033** |
| Admission to NICU |  |  |  |  |  |  |
| Model 1 | 1.74 (1.41–2.15) | Ref. | 1.36 (0.63–3.00) | 2.02 (1.00–4.29) | 4.18 (2.21–8.52) | **<0.001** |
| Model 2 | 1.70 (1.37–2.12) | Ref. | 1.29 (0.60–2.87) | 1.95 (0.96–4.16) | 3.84 (2.01–7.87) | **<0.001** |
| Model 3 | 1.66 (1.31–2.10) | Ref. | 1.13 (0.51–2.54) | 1.56 (0.74–3.42) | 3.13 (1.56–6.66) | **<0.001** |

**Note:** Model 1 was unadjusted. Model 2 was Model 1 adjusted for age, pre-pregnancy BMI, and parity. Model 3 was Model 2 adjusted for TC, LDL-C, OGTT-FPG, and HbA1c. The bold *P* value indicates statistical significance.

**Abbreviations:** AIP, atherogenic index of plasma; GDM, gestational diabetes mellitus; NICU, neonatal intensive care unit; PE, preeclampsia; Q, quartiles.

Supplementary Table 17. Associations of cumulative AIP with maternal and neonatal outcomes in Validation cohort 2.

|  | Continuous | Cumulative AIP quartiles | | | | *P* for trend |
| --- | --- | --- | --- | --- | --- | --- |
|  |  | Q1 | Q2 | Q3 | Q4 |  |
| PE |  |  |  |  |  |  |
| Model 1 | 2.50 (1.95–3.24) | Ref. | 5.73 (1.52–37.30) | 4.64 (1.18–30.68) | 21.19 (6.36–131.45) | **<0.001** |
| Model 2 | 2.24 (1.72–2.94) | Ref. | 5.41 (1.42–35.37) | 3.85 (0.97–25.61) | 16.00 (4.74–99.86) | **<0.001** |
| Model 3 | 2.28 (1.72–3.05) | Ref. | 5.76 (1.48–38.11) | 4.16 (1.01–28.21) | 17.34 (4.80–111.92) | **<0.001** |
| PE with severe features * |  |  |  |  |  |  |
| Model 1 | 2.96 (2.03–4.44) | Ref. | 5.04 (0.41–697.22) | 5.04 (0.41–697.22) | 30.89 (4.09–3956.74) | **<0.001** |
| Model 2 | 2.49 (1.65–3.84) | Ref. | 4.75 (0.38–657.74) | 4.27 (0.34–590.94) | 21.49 (2.78–2765.32) | **<0.001** |
| Model 3 | 2.80 (1.76–4.59) | Ref. | 4.81 (0.38–669.79) | 4.46 (0.34–624.84) | 23.42 (2.73–3079.82) | **<0.001** |
| Admission to NICU |  |  |  |  |  |  |
| Model 1 | 1.62 (1.33–1.98) | Ref. | 1.54 (0.73–3.36) | 2.22 (1.11–4.68) | 3.71 (1.94–7.60) | **<0.001** |
| Model 2 | 1.58 (1.28–1.95) | Ref. | 1.50 (0.71–3.29) | 2.04 (1.01–4.34) | 3.39 (1.75–7.00) | **<0.001** |
| Model 3 | 1.53 (1.22–1.92) | Ref. | 1.27 (0.59–2.82) | 1.64 (0.79–3.57) | 2.76 (1.36–5.92) | **0.002** |

**Note:** Model 1 was unadjusted. Model 2 was Model 1 adjusted for age, pre-pregnancy BMI, and parity. Model 3 was Model 2 adjusted for TC, LDL-C, OGTT-FPG, and HbA1c. The bold *P* value indicates statistical significance. Firth's bias-reduced logistic regression was applied due to complete separation in the first quartile group of PE with severe features.

**Abbreviations:** AIP, atherogenic index of plasma; GDM, gestational diabetes mellitus; NICU, neonatal intensive care unit; PE, preeclampsia; Q, quartiles.

Supplementary Table 18. Receiver operating characteristic curve analysis of AIP for detecting GDM-related maternal and neonatal outcomes in Validation cohort 2.

|  | Variables | AUC (95% CI) | Best threshold | Specificity | Sensitivity | Youden index | *P* value |
| --- | --- | --- | --- | --- | --- | --- | --- |
| PE | AIP | 0.700 (0.630–0.769) | 0.026 | 0.540 | 0.793 | 0.333 |  |
|  | Clinical variables | 0.700 (0.627–0.774) | 0.073 | 0.714 | 0.655 | 0.369 |  |
|  | Combined | 0.762 (0.704–0.819) | 0.061 | 0.689 | 0.724 | 0.413 | **0.036** |
| PE with severe features | AIP | 0.762 (0.640–0.885) | 0.593 | 0.743 | 0.722 | 0.465 |  |
|  | Clinical variables | 0.679 (0.534–0.825) | 0.029 | 0.814 | 0.556 | 0.370 |  |
|  | Combined | 0.785 (0.673–0.898) | 0.025 | 0.804 | 0.667 | 0.471 | **0.035** |
| Admission to NICU | AIP | 0.659 (0.601–0.717) | 0.205 | 0.628 | 0.638 | 0.267 |  |
|  | Clinical variables | 0.649 (0.592–0.706) | 0.084 | 0.428 | 0.798 | 0.226 |  |
|  | Combined | 0.695 (0.642–0.749) | 0.104 | 0.630 | 0.681 | 0.310 | **0.044** |

**Note:** Clinical variables included age, pre-pregnancy BMI, parity, TC, LDL-C, OGTT-FPG, and HbA1c. The *P* value indicates differences between clinical variables AUC and AIP + clinical variables (combined) AUC. The bold *P* value indicates statistical significance.

**Abbreviations:** AIP, atherogenic index of plasma; AUC, area under the curve; GDM, gestational diabetes mellitus; NICU, neonatal intensive care unit; PE, preeclampsia.

Supplementary Table 19. Receiver operating characteristic curve analysis of cumulative AIP for detecting GDM-related maternal and neonatal outcomes in Validation cohort 2.

|  | Variables | AUC (95% CI) | Best threshold | Specificity | Sensitivity | Youden index | *P* value |
| --- | --- | --- | --- | --- | --- | --- | --- |
| PE | Cumulative AIP | 0.755 (0.691–0.819) | 0.642 | 0.800 | 0.621 | 0.420 |  |
|  | Clinical variables | 0.700 (0.627–0.774) | 0.073 | 0.714 | 0.655 | 0.369 |  |
|  | Combined | 0.789 (0.737–0.842) | 0.035 | 0.540 | 0.897 | 0.436 | **0.002** |
| PE with severe features | Cumulative AIP | 0.852 (0.764–0.940) | 0.803 | 0.822 | 0.778 | 0.600 |  |
|  | Clinical variables | 0.679 (0.534–0.825) | 0.029 | 0.814 | 0.556 | 0.370 |  |
|  | Combined | 0.850 (0.755–0.945) | 0.020 | 0.794 | 0.778 | 0.572 | **<0.001** |
| Admission to NICU | Cumulative AIP | 0.648 (0.589–0.706) | 0.255 | 0.656 | 0.574 | 0.231 |  |
|  | Clinical variables | 0.649 (0.592–0.706) | 0.084 | 0.428 | 0.798 | 0.226 |  |
|  | Combined | 0.692 (0.638–0.747) | 0.078 | 0.468 | 0.830 | 0.298 | **0.036** |

**Note:** Clinical variables included age, pre-pregnancy BMI, parity, TC, LDL-C, OGTT-FPG, and HbA1c. The *P* value indicates differences between clinical variables AUC and AIP + clinical variables (combined) AUC. The bold *P* value indicates statistical significance.

**Abbreviations:** AIP, atherogenic index of plasma; AUC, area under the curve; GDM, gestational diabetes mellitus; NICU, neonatal intensive care unit; PE, preeclampsia.


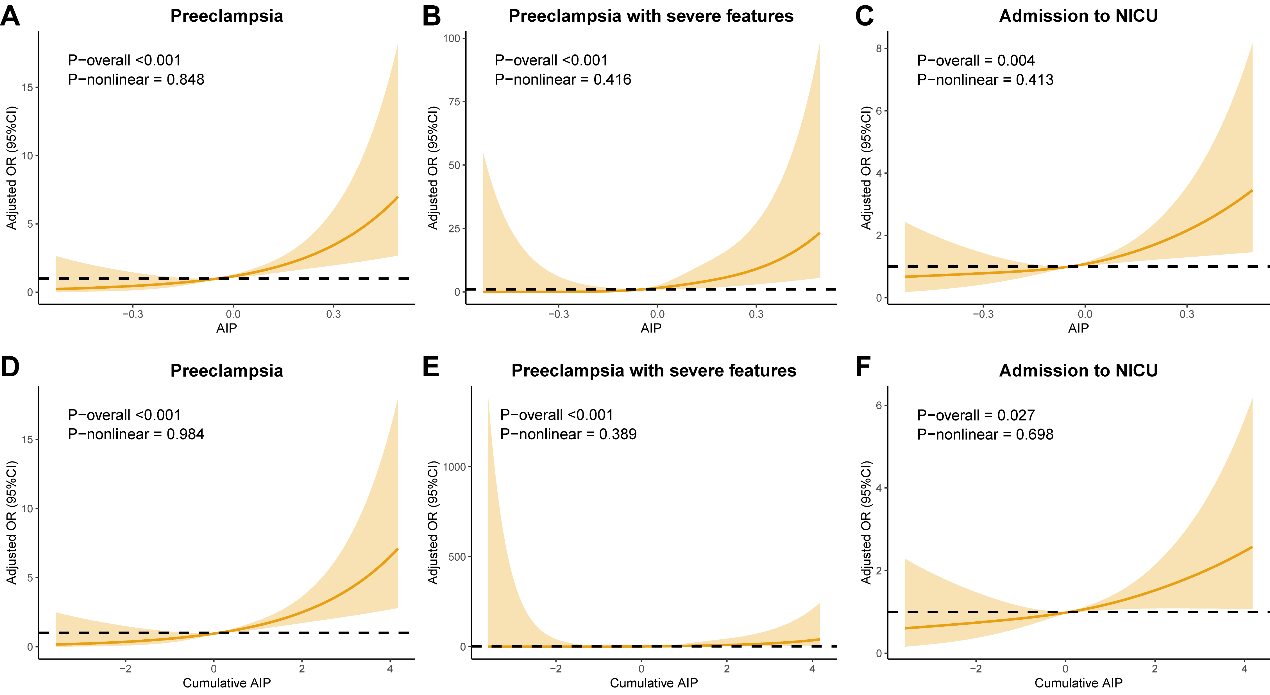


Supplementary Fig. 1. Multivariate RCS regression analysis for the nonlinear association of AIP and cumulative AIP with GDM-related maternal and neonatal outcomes. **A** Preeclampsia. **B** Preeclampsia with severe features. **C** Admission to NICU. **D** Preeclampsia. **E** Preeclampsia with severe features. **F** Admission to NICU. Models were adjusted for age, pre-pregnancy BMI, parity, TC, LDL-C, OGTT-FPG, and HbA1c. AIP, atherogenic index of plasma; GDM, gestational diabetes mellitus; NICU, neonatal intensive care unit; RCS, restricted cubic spline.


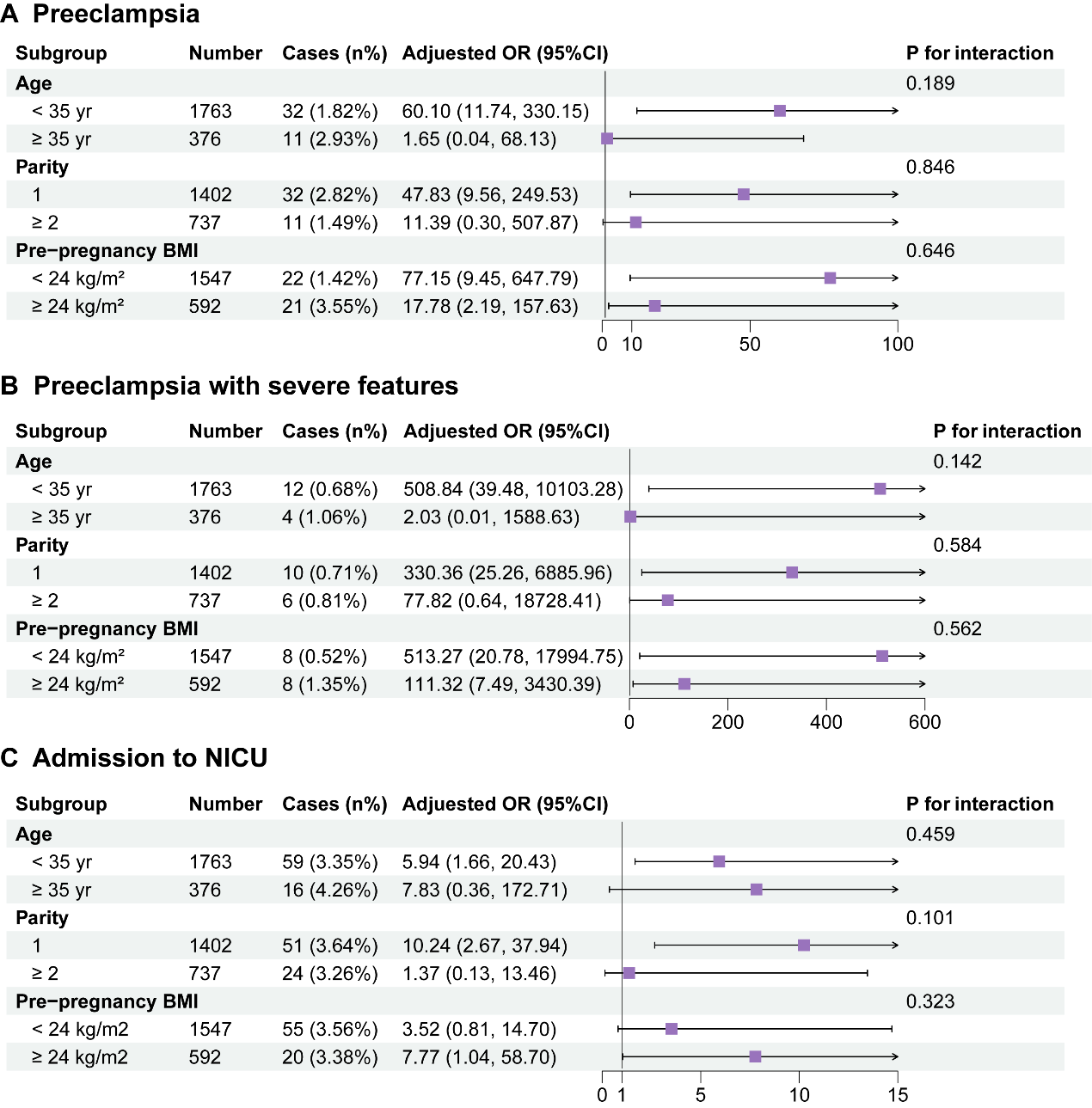


Supplementary Fig. 2. Subgroup analysis for the association of AIP with GDM-related maternal and neonatal outcomes. **A** Preeclampsia. **B** Preeclampsia with severe features. **C** Admission to NICU. Models were adjusted for age, pre-pregnancy BMI, parity, TC, LDL-C, OGTT-FPG, and HbA1c, except the stratification factor itself. AIP, atherogenic index of plasma; BMI, body mass index; GDM, gestational diabetes mellitus; NICU, neonatal intensive care unit; y, year.


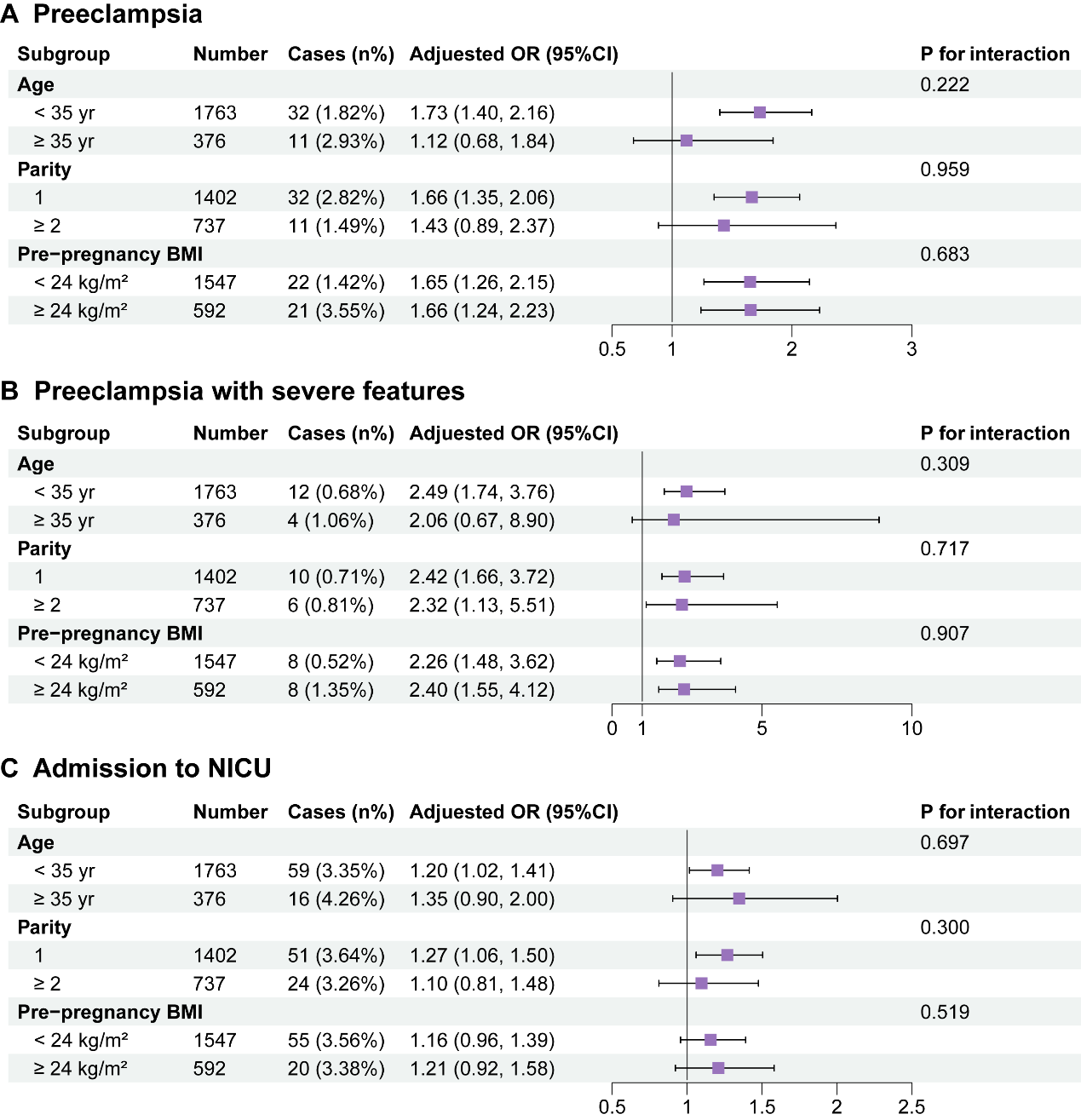


Supplementary Fig. 3. Subgroup analysis for the association of cumulative AIP with GDM-related maternal and neonatal outcomes. **A** Preeclampsia. **B** Preeclampsia with severe features. **C** Admission to NICU. Models were adjusted for age, pre-pregnancy BMI, parity, TC, LDL-C, OGTT-FPG, and HbA1c, except the stratification factor itself. AIP, atherogenic index of plasma; BMI, body mass index; GDM, gestational diabetes mellitus; NICU, neonatal intensive care unit; y, year.
